# Anti-oxidant and anti-inflammatory potential of aqueous extracts of leaves, barks and roots of *Bixa orellana* L. (Bixaceae) on acetaminophen-induced liver damage in mice

**Published:** 2020

**Authors:** David Gatsou Djibersou, Borris Rosnay Tietcheu Galani, Pascal Dieudonne Djamen Chuisseu, Nicolas Yanou Njintang

**Affiliations:** 1 *Laboratory of Applied Biochemistry, Department of Biological Sciences, Faculty of Science, University of Ngaoundere, PO Box 454 Ngaoundere, Adamawa, Cameroon*; 2 *Basic Science Department, Faculty of Health Sciences, Université des Montagnes, PO Box 208 Bangangté, West, Cameroon*

**Keywords:** Bixa Orellana, Acetaminophen, Hepatotoxicity, Antioxidant, Mice

## Abstract

**Objective:**

*Bixa orellana *is a plant from the Bixaceae family, for which, limited information is available on hepatoprotective properties. This study aimed at evaluating the protective effects of this plant on sub-acute acetaminophen (APAP)-induced liver injury in mice.

**Materials and Methods:**

Various aqueous extracts were prepared from roots, leaves, and barks. Albino mice were divided into six groups: a control group, an APAP group; a silymarin group (positive control) and three test groups. Mice were treated orally with APAP (250 mg/kg) followed 3 hr later by plant extracts, silymarin (50 mg/kg) or distilled water (10 ml/kg) administration once daily, for seven days. After treatment, animals were sacrificed, the liver was collected and different biochemical parameters were measured. Histological analyses were performed using hematoxylin/eosin staining and the qualitative phytochemical content of plant extracts was evaluated using conventional methods.

**Results:**

Administration of *B. orellana* barks decoction (250 mg/kg) significantly reduced alanine aminotransferase levels (p<0.001), unlike leaves and roots extracts. Moreover, the bark infusion had the highest activity compared to macerate and decoction. It significantly reduced malondialdehyde levels (p<0.001) and increased the levels of glutathione, superoxide dismutase and catalase, at doses of 250 and 500 mg/kg compared to the APAP group. A significant (p<0.001) decrease of tumor necrosis factor-α levels and leukocyte infiltration was found following treatment with bark infusion. The infusion content evaluation revealed the presence of polyphenols, saponins, tannins, sterols, anthraquinones, and coumarins and the absence of alkaloids.

**Conclusion:**

These results show that infusion from *B. orellana* barks is hepatoprotective against APAP-induced toxicity via antioxidant and anti-inflammatory mechanisms.

## Introduction

The liver is the largest internal organ in vertebrates and some other animals. It controls and regulates a wide variety of biochemical reactions including the biotransformation and elimination of xenobiotics, making it more sensitive to the toxicity of these agents (Fortner and Blumgart, 2001 ▶). Acetaminophen (N-acetyl-para-aminophenol; APAP), also known as paracetamol, is the active substance of many drugs belonging to the class of non-salicylate antipyretic analgesics. It is available by prescription or over-the-counter for headaches, pain, allergies, and colds, (El-Bahri, 2015 ▶). Currently, it is a leader in antipyretic analgesics. First competing with aspirin, its global consumption is much higher today because its therapeutic dose (3000 to 4000 mg/day in adults) is easily tolerated. According to some studies, APAP toxicity would be responsible for 46% of cases of acute liver failure in the United States and about 70% in Europe (Lee, 2017 ▶). 

APAP is known to be safe at therapeutic doses with analgesic and antipyretic effects similar to those of ibuprofen or aspirin (Hinson et al., 2010 ▶). At these doses (3000 to 4000 mg/day in adults), APAP is mainly transformed into non-toxic metabolites through reactions of glucuronidation or sulfuration. However, a small amount is metabolized by cytochrome P450 enzymes to form a toxic derivative, N–acetyl–p–benzoquinone imine (NAPQI) which is rapidly conjugated with glutathione (GSH). Nonetheless, at higher doses, NAPQI accumulates in the liver and leads to GSH depletion. This situation will cause oxidative stress and induce a centrilobular hepatic necrosis that may be lethal (Hinson et al., 2010 ▶). After this initial liver necrosis, resident macrophages will be activated by molecular signals and will produce pro-inflammatory chemokines and cytokines which will result in the infiltration and activation of neutrophils and macrophages and exacerbate the liver injury (Lee et al., 2019 ▶). 

Medicinal plants are widely used for the prevention and treatment of various diseases worldwide and particularly in developing countries (Keppler and Decker, 1969 ▶). They are now sources of natural substances used in the treatment of many conditions among which are liver diseases. Silymarin, isolated from *Silybum marianum, *is a flavonolignan used for hepatoprotection due to its antioxidant, antifibrotic and anti-inflammatory activities (Polyak et al., 2010 ▶). Previous studies reported that silymarin prevents APAP hepatotoxicity in mice through antioxidant mechanisms (Papackova et al., 2018 ▶). Therefore, this compound is used as a reference standard in experimental studies (Mahmoud et al., 2014 ▶). *Bixa orellana*, a plant of the Bixaceae family, native to Brazil, is grown in tropical countries such as Peru, Mexico, Ecuador, Malaysia, Indonesia, India, Kenya and East Africa (Elias et al., 2002 ▶). Plant roots and leaves are used as diuretic and antipyretic agents in the management of sore throat, jaundice, dysentery, gonorrhoea and liver diseases (Castello et al., 2002 ▶). The annatto dye extracted from its seeds is used in coloring butter, margarine, cheese, beverages and meat and fish products (Radhika et al., 2010 ▶). Many pharmacological properties have been reported for extracts from different parts of this plant, including antimicrobial (Galindo-cuspinera et al., 2003 ▶), anthelmintic (Padhi and Panda, 2016 ▶; Karmakar et al., 2018 ▶), antioxidant (Abayomi et al., 2014 ▶), antigenotoxic and antimutagenic (Kovary et al., 2005 ▶), diuretic (Radhika et al., 2010 ▶), anticonvulsant (Panda et al., 2018 ▶), hypoglycemic (Russell et al., 2008 ▶), anti-inflammatory (Keong et al., 2011 ▶), anti-histaminic (Yong et al., 2013 ▶) and hepatoprotective activities (Bell et al., 2012 ▶; Lopez et al., 2017 ▶; Singh et al., 2018 ▶). An earlier study of the bark extract of *B. orellana* reported an antioxidant activity in the liver after a chronic APAP exposure (Bell et al., 2012 ▶). However, the effects of this plant on a short-term toxicity remain unknown. In the current study, the hepatoprotective effects of different aqueous extracts of *B. orellana* were investigated against sub-acute APAP-induced liver injury in mice.

## Materials and Methods

**Chemical and reagents**

The standard treatment used in this work, was silymarin (140 mg tablet), dissolved in distilled water and administered to animals at a dose of 50 mg/kg. The hepatotoxin used was APAP tablet (Doliprane 500 mg). These drugs (APAP and silymarin) manufactured by Sanofi-Aventis (Longjumeau, France) and Micro Labs Limited (Hosur, India) respectively, were purchased at the Pharmacy of SARE, in Ngaoundéré, Cameroon. The alanine aminotransferase (ALT) and tumor necrosis factor alpha (TNF-α) kits used for the assays were from Linear Chemicals .S.L. Joaquim Costa 18 2a planta (Barcelona, Spain) and Solarbio Life Sciences (Beijing, China), respectively.

**Plant material**

The plant material consisted of the roots, leaves and barks of *B. orellana*, harvested on the campus of the University of Ngaoundéré, in the Vina Division of the Adamawa Region of Cameroon. Samples were collected on August 10, 2018, very early in the morning between 6 and 10 am. The botanical identification of the plant was made by Prof. Mapongmetsem Pierre Marie, a botanist at the Faculty of Sciences of the University of Ngaoundéré, Cameroon. After harvest, plant samples were washed with tap water to remove dirt, cut into small pieces to facilitate drying, dried in the shade for 3 weeks, then crushed and sieved through a mesh of pore size 695 μm in diameter, to obtain powders.

**Preparation of aqueous extracts**

Twenty-five (25) g of the *B. orellana* bark powder was weighed and mixed with 250 ml of distilled water. For preparing the decoction, the mixture was boiled at 100°C for 20 min. For the infusion, powder was mixed with boiling water for 20 min, while for macerate, the powder was just mixed with distilled water for 20 min at room temperature. After cooling, the mixtures were filtered and the filtrates were frozen and lyophilized at -55°C. A yield of 4.68% was obtained for the decoction, 5.92% for the infusion and 6.28% for the macerate. The freeze-dried extracts were packed in aluminum foil and stored in boxes at 4°C until use. 

**Phytochemical analyses**

Plant samples were analyzed using conventional methods to determine the existence of alkaloids, flavonoids, polyphenols, tannins, saponins, coumarins and anthraquinones, as previously reported (Wadood et al., 2013 ▶).

**Experimental animals and treatments**

Albino mice (*Mus musculus*) aged 2 to 3 months, weighing 18 to 35 g, were used. Animals were provided by the pet store of the National School of Agro-Industrial Sciences of the University of Ngaoundéré (Cameroon) and placed in plexiglass cages containing chips, provided with a steel grid preventing their exit. Mice had free access to food and tap water and were kept at room temperature under conditions of the animal facility. Mice were used according to the Animal Research: Reporting *in vivo* Experiments (ARRIVE) guidelines and permission for experiments was given by the Animal Ethics Committee of the Faculty of Science of the University of Ngaoundéré (200/2019/UN/DFS/VD-RC/CSPDR).

In order to evaluate the effects of extracts from different parts of the plant on APAP hepatotoxicity, a first experiment was conducted. Thirty (30) mice were divided into six groups of 5 mice as follows: Group I: normal control (NC) received 10 ml/kg of distilled water; Group II: negative control (APAP) treated with 250 mg/kg of APAP (Lee, 2017 ▶); Group III: positive control treated with 250 mg/kg APAP and 50 mg/kg silymarin (Karimi et al., 2011 ▶); Group IV: test groups, containing three batches of 5 mice each, received 250 mg/kg of APAP and 250 mg/kg of the decoction of leaves, barks, and roots, respectively. The choice of doses was made based on previous studies (De-Oliveira et al., 2003 ▶). Hepatotoxicity was induced by administering 250 mg/kg APAP to mice , except the normal control mice that received distilled water. The plant extract and silymarin were administered 3 hr after paracetamol treatments once a day for one week. All treatments were administered orally via a gastric tube.

At the end of this experiment, the most active extract was identified and different modes of its preparation were tested to determine the most efficient one. Then, mice were grouped and treated as mentioned above. However, in Group IV, the three batches received 250 mg/kg of APAP and 250 mg/kg of decoction, macerate or infusion from the most effective plant part. Treatments lasted 7 days and on the 8^th ^day, animals were sacrificed and the livers were collected, crushed with a mortar and pestle and centrifuged at 4000 rpm for 10 min at 4 ° C. ALT levels were then measured in liver samples. At the end of this test, the method of preparation, having shown the most effective hepatoprotective effect, was retained for further investigations. 

Seven batches of 5 mice each, were used in this third test as follows: Groups I, II and III were treated as mentioned above. Group IV was divided into four batches. The first three received 250 mg/kg of APAP and 3 hr later, 100, 250 and 500 mg/kg of the bark’s infusion was administrated *per os* respectively to each batch. The fourth batch received only 500 mg/kg of bark’s infusion and no APAP, to test the extract toxicity. All four batches were treated daily for one week. Thereafter, animals were sacrificed, the liver homogenates were prepared and the biochemical parameters were measured. 

**Preparation of liver tissue homogenates**

Upon dissection, liver samples were collected for the preparation of liver homogenates. A portion of the liver was ground in a mortar, then 1 ml of the ground material was homogenized in 9 ml (1/10) volumes of 0.1 M phosphate buffer, pH 7.4. The homogenate was centrifuged at 10,000×g for 15 min at -4°C and aliquots of supernatant were used for biochemical estimations.

**Assessment of markers of liver function**

ALT levels were determined as previously described (Reitman and Frankel, 1957 ▶) using the commercial detection kits according to the manufacturer's instructions.

**Assessment of oxidative stress parameters**

Activities of catalase (CAT) and superoxide dismutase (SOD) were assessed as antioxidant parameters using commercial detection kits (Sigma-Aldrich Chemical Company, USA) according to the manufacturer's instructions. The degree of lipid peroxidation was measured by quantifying malondialdehyde (MDA) levels as previously reported (Ohkawa et al., 1979 ▶) and the reduced glutathione assay (GSH) was performed according to the protocol described previously (Ellman, 1959 ▶).

**Quantification of TNF-α expression in liver homogenates**

TNF-α levels in liver homogenates were measured by sandwich ELISA using a commercial kit from Solarbio Life Sciences (Beijing, China).

**Histopathological examination**

The liver tissue was fixed in 4% paraformaldehyde, embedded in paraffin, sectioned into 5 μm thickness, and stained with hematoxylin and eosin (H&E) for evaluation of histopathological changes. Microscopic observations were performed under a bright-field microscope. 

Different pathological grades were considered in order to evaluate the severity of inflammation. Grade 0 (no pathological change), grade 1 (minimal leukocyte infiltration), grade 2 (mild), grade 3 (moderate), grade 4 (important leukocyte infiltration) (Takahashi and Fukusato, 2014 ▶).

**Statistical analysis**

The results are expressed as mean±SEM. Data were analyzed by a single-way analysis of variance (ANOVA) followed by a Dunnett Multiple Comparisons test, using GraphPad prism5.0 software. A difference was considered statistically significant at p<0.05.

## Results

**Effect of different parts of** ***Bixa orellana*** **on APAP-induced liver injury**

**Figure 1:**
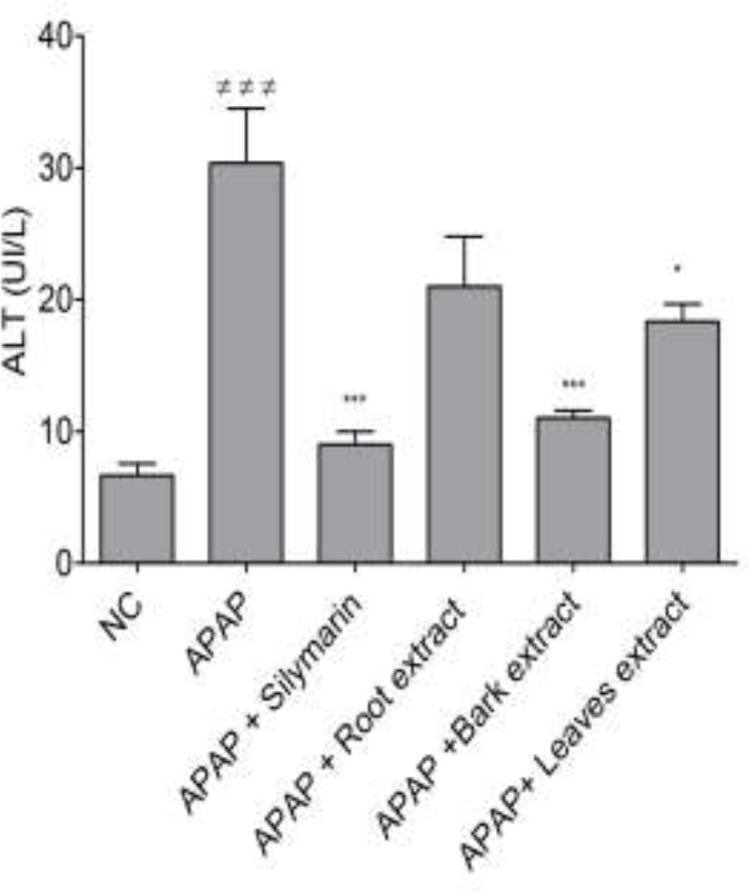
Effect of the decoction of roots, barks and leaves of* B. orellana* on ALT levels in APAP hepatotoxicity. NC=normal control; ME=macerate; IE=infusion; DE=decoction; and ≠≠≠ p<0.001 when compared to NC group. *p<0.05 and ***p<0.001 show significant differences when compared to the APAP group

Figure 1 presents the effects of the decoction of roots, barks, and leaves of* B. orellana *on ALT levels in APAP-induced hepatotoxicity. According to this Figure, the bark and leaf extracts at 250 mg/kg exhibited a significant (p*<*0.001 and p<0.05 respectively) reduction in ALT levels while the reduction induced by the root extract was not significant. Of these 3 extracts, the bark had the most pronounced reduction in ALT levels, similar to that of silymarin used as the positive control.

**Effect of the mode of preparation of** ***Bixa orellana*** **bark extracts on ALT levels**

**Figure 2.**
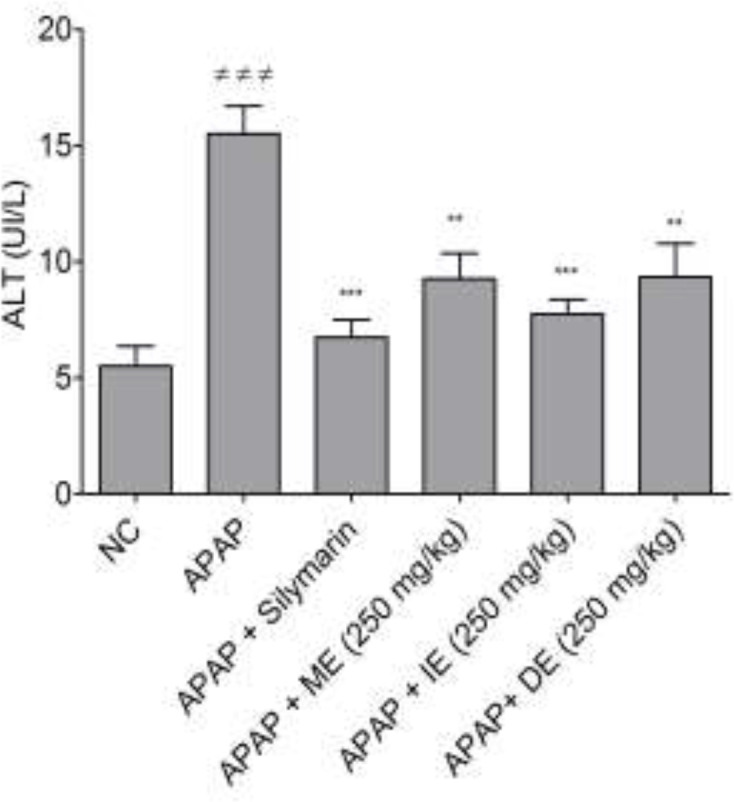
Effect of the mode of preparation of aqueous barks extracts of* B. orellana* on ALT levels in APAP hepatotoxicity. NC=normal control; ≠≠≠p<0.001 shows significant differences when compared to the NC group. **p<0.01 and ***p<0.001 show significant differences when compared to the APAP group

Figure 2 shows the effects of barks infusion (IE), decoction (DE), and macerate (ME) on ALT activity in treated mice. All the aqueous extracts significantly (p<0.01) lowered the ALT levels compared to the negative control. Moreover, the infusion had the lowest ALT levels compared to other groups (decoction and macerate), similar to the effect of silymarin. This decrease suggests that the bark’s infusion has a more marked hepato-protective effect than the macerate, and decoction when tested at 250 mg/kg.

**Effect of bark’s infusion on ALT levels**

**Figure 3:**
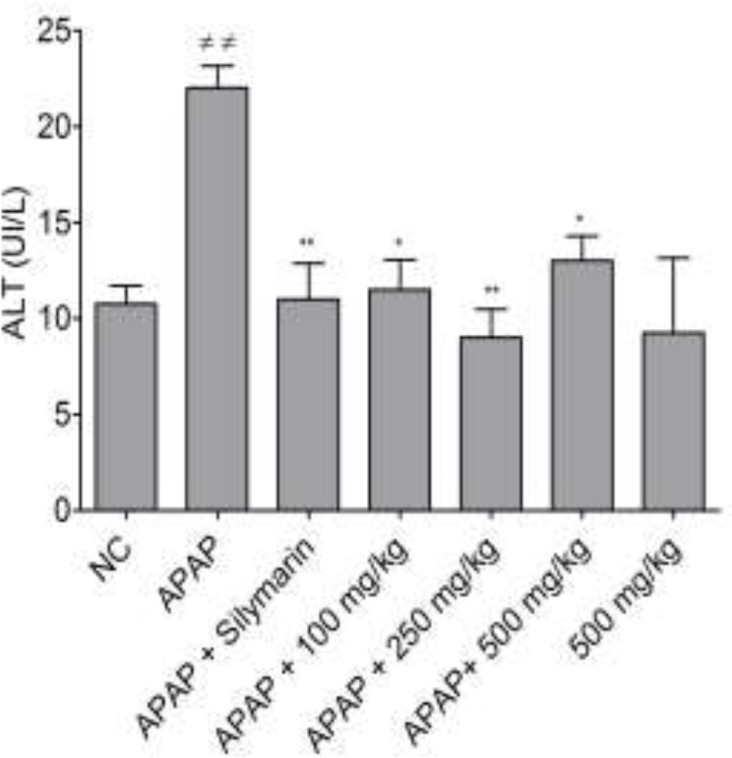
Effect of different doses of the bark’s infusion on ALT levels in APAP hepatotoxicity. NC=normal control; ≠≠p<0.01 shows significant differences when compared to the NC group. *p<0.05 and **p<0.01 show significant differences when compared to the APAP group

Figure 3 shows the effect of different doses of the bark’s infusion of* B. orellana *on ALT levels in the liver of intoxicated mice. All the tested doses significantly alleviated ALT levels compared to the negative control (p<0.05 and p<0.01). Similarly, a significant decrease was found (p<0.01) in the positive control compared to the negative control. For the groups of mice that received only plant extract at 500 mg/kg, there was no significant difference compared to the normal control, suggesting the safety of this plant extract.

**Effect of the bark’s infusion on oxidative stress markers**

**Figure 4:**
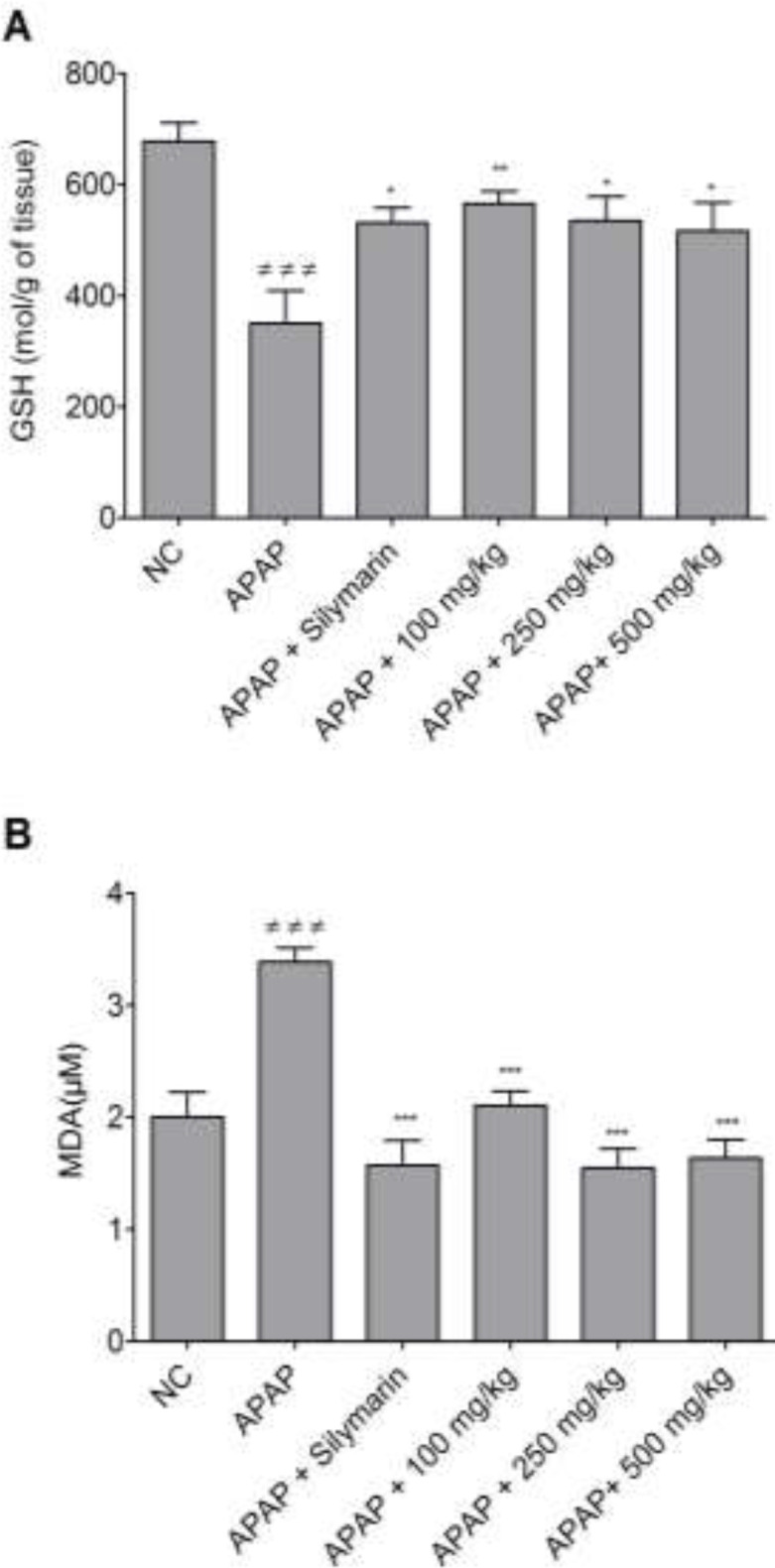
Effect of different doses of the bark’s infusion of *B. orellana* on GSH (a) and MDA (b) levels in liver homogenates of mice treated with APAP. NC=normal control; ≠≠≠ p<0.001 shows significant differences when compared to the NC group. *p<0.05, **p<0.01 and ***p<0.001 show significant differences when compared to the APAP group

Figure 4 illustrates the effect of bark’s infusion on GSH and MDA levels in animal liver homogenates. It shows that the GSH levels in APAP-treated mice significantly decreased compared to the normal control (Figure 4A). The administration of the bark’s infusion, significantly increased the GSH levels at all tested doses compared to the APAP group, showing the capacity of the plant extract to reconstitute the GSH stock depleted by NAPQI. In Figure 4B, APAP-treated mice showed significantly increased MDA values (p<0.001) compared to the normal control group. Administration of the bark’s infusion significantly decreased (p<0.001) these values compared to the APAP group. This result demonstrates that the bark’s infusion of *B. orellana *inhibits lipid peroxidation in the liver. The effect of bark’s infusion of *B. orellana* on the activity of CAT and SOD was also evaluated (Table 1). In APAP-treated mice, the activity of CAT and SOD decreased significantly (p<0.01) compared to the normal control. Administration of the bark’s infusion significantly inhibited enzymes activity compared to the APAP group.

**Table 1:** Effect of *Bixa orellana* bark’s infusion on superoxide dismustase (SOD) and catalase (CAT) activities in liver homogenates

**Group**	**SOD (UI/ml)**	**CAT (μl/ml)**
NC	98.08±10.67	510.93±46.85
APAP 250 mg/kg	41.61±2.34^≠≠^	338.42±20.34^≠≠^
APAP + 50 mg/kg silymarin	78.10±6.88**	486.28±20.15**
APAP + 100 mg/kg infusion	53.43±7.09*	486.28±46.93**
APAP + 250 mg/kg infusion	90. 86±9.06**	658.80±45.58**
APAP + 500 mg/kg infusion	68.34±8.76**	517.54±52.18**

NC=normal control; ≠≠ p<0.01 shows significant differences when compared to the NC group. *p<0.05 and **p<0.01 show significant differences when compared to the APAP group.

**Effect of the bark’s infusion of** ***Bixa orellana*** **on TNF-α expression**

**Figure 5:**
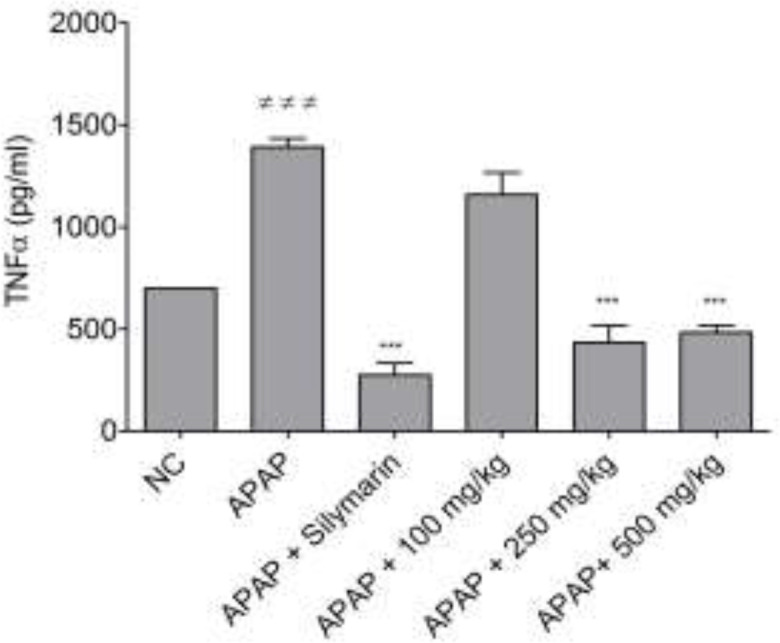
Effect of the *B. orellana* bark’s infusion on TNF-α levels in liver homogenates of APAP-treated mice. NC=normal control ≠≠≠p<0.001 shows significant differences when compared to the NC group. ***p<0.001 shows significant differences when compared to the APAP group

Inflammation and oxidative stress are two important processes during APAP-induced hepatitis. Since bark’s infusion was found to interfere with oxidative stress, we investigated its effects on the pro-inflammatory mediator, TNF-α. Figure 5 shows the effect of bark’s infusion on TNF-α secretion in liver homogenates. TNF-α expression in mice significantly increased (by 2-fold range) in the APAP-treated group compared to the non-treated group (NC). The silymarin treatment showed a significant decrease (p<0.001) of TNF-α levels at the dose tested (50 mg/kg). Similarly, in the mice treated with the bark’s infusion, a significant (p<0.001) reduction of TNF-α values was observed at the 250 and 500 mg/kg doses. However, at 100 mg/kg, this decrease was not statistically significant. 

**Effects of the bark’s infusion of** ***Bixa orellana*** **on histological sections**

**Figure 6:**
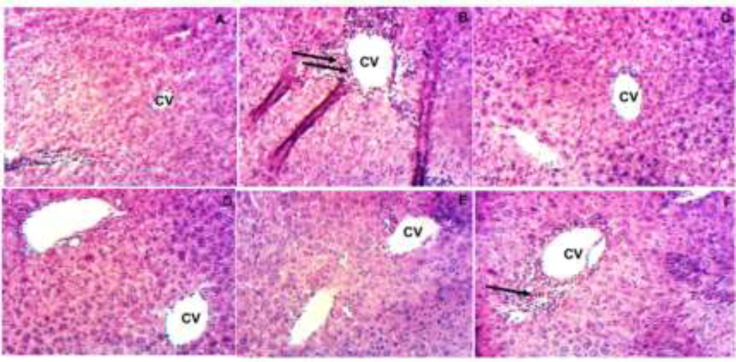
Representative micrographs of liver sections of mice treated with different doses of the bark’s infusion of *B. orellana* (magnification X 100). A: normal control; B: APAP-treated mice. Black arrows show high leukocyte infiltration around the centrilobular vein (CV); C: 250 mg/kg APAP+Silymarin (50 mg/kg); D: (250 mg/kg APAP+bark’s infusion (100 mg/kg)); E: (250 mg/kg APAP+bark’s infusion (250 mg/kg)); and F: (250 mg/kg APAP+bark’s infusion (500 mg/kg)).

To confirm the necroinflammatory activity on liver tissue, the effect of bark’s infusion was also examined by H&E staining. As shown in Figure 6, APAP administration (Figure 6B), resulted in an important infiltration of the inflammatory cells around the centrilobular vein, with hepatic necrosis compared to the normal group (Figure 6A) where no pathological change was observed. In the positive control (Figure 6C) and test groups, there was a structural improvement in the liver at 100 and 250 mg/kg (Figures 6D and E) evidenced by a significant decrease of the pathological score as compared to the APAP (Table 2). However, with bark infusion at 500 mg/kg, a moderate infiltration of leukocytes was observed suggesting a partial restoration of liver tissue (Figure 6F).

**Phytochemical profile**

The qualitative phytochemical analysis of the different aqueous extracts showed some differences in the composition of macerate, infusion, and bark decoction. The infusion contained most of the metabolites tested, except alkaloids, while only saponins were missing from the bark macerate, and flavonoids and alkaloids were absent in bark decoction (Table 3).

**Table 2:** Effects of the *Bixa orellana* bark’s infusion on the liver histopathological grades induced by sub-acute APAP administration

**Group**	**N**	**Histopathological score**
NC	5	0.0±0.0
APAP 250 mg/kg	5	3.5±0.6^≠^^≠^
APAP+Silymarin 50 mg/kg	5	1.2 ±0.3**
APAP+100 mg/kg infusion	5	1.0±0.2**
APAP+250 mg/kg infusion	5	0.6±0.4**
APAP+500 mg/kg infusion	5	2.1 ±0.2**

Results are presented as mean±SEM. ^≠^^≠^p*<*0.01 shows significant differences compared to the normal control (NC) **p*<*0.01 shows significant differences compared to the APAP group. n=5; comparisons made by one-way ANOVA and Dunnett *post hoc* test.

**Table 3:** Qualitative phytochemical profile of the different aqueous extracts of *Bixa orellana*

Aqueous extracts	Decoction			Barks Macerate	Barks infusion
	Leaves	Barks	Roots		
Polyphenols	+	+	+	+	+
Alkaloids	+	-	+	+	-
Flavonoids	+	-	+	+	+
Saponins	-	+	+	-	+
Tannins	+	+	+	+	+
Sterols	+	+	+	+	+
Anthraquinones	+	+	+	+	+
Coumarins	+	+	+	+	+

## Discussion

The liver is an essential organ in many biological functions including the metabolism of carbohydrates, lipids, and proteins, the regulation of blood clotting, the cholesterol transportation, resistance against infections, in addition to metabolism and clearance of xenobiotics (Fortner and Blumgart, 2001 ▶). Therefore, it is primarily affected by toxic agents. Alteration of liver functions can be assessed by several biochemical markers including transaminases, total bilirubin, alkaline phosphatase and albumin (Ahmed et al., 2018 ▶). Plant extracts can play an important role in preventing liver damage.

APAP is the most commonly used analgesic and antipyretic medication. Taking this drug at excessive doses generates a reactive compound NAPQI, that may accumulate in the liver and therefore lead to antioxidant depletion or trigger hepatic cytolysis (Hinson et al., 2010 ▶). *Bixa orellana*, a plant of the family of Bixaceae, was reported to possess protective activities against carbon tetrachloride- and ethanol-induced hepatotoxicity, but the effects on APAP were poorly studied. In this work, we evaluated *in vivo* hepatoprotective activities of different aqueous extracts of this plant on APAP-induced liver injury. A first screening of the bioactivity of roots, leaves, and barks aqueous extracts showed a more pronounced inhibition with the bark extract, suggesting, a concentration of active metabolites in this plant part compared to other parts. Further investigations with aqueous extracts from the bark revealed a significant effect with the infusion compared to decoction and macerate, demonstrated by the decrease of ALT levels. This result might indicate that heat played a role in the extraction process of active ingredients as reported previously (Chavan et al., 2001 ▶). Moreover, this activity could be also correlated with the nature of secondary metabolites contained in each sample. As shown in Table 3, the lack of alkaloids and the presence of flavonoids in the bark infusion seemed to have played a crucial part in this extract's bioactivity. In fact, contrary to flavonoids, which are mainly known to be very bioactive compounds, some alkaloids could possess liver toxic effects as previously reported (Kovary et al., 2005 ▶). The findings of this research are consistent with those of Sabir and Rocha who attributed *Solanum fastigiatum* hepatoprotective activities to the presence of flavonoids in the water extract (Sabir and Rocha, 2008 ▶). Investigating the effects of plant extracts on oxidative stress parameters showed that MDA, CAT, SOD and GSH levels were significantly affected by APAP treatment. This may be interpreted as a consequence of liver cell destruction or changes in membrane permeability indicating hepatocellular damage caused by APAP. However, treatment with the barks infusion attenuated these changes in liver homogenates, suggesting, therefore, an antioxidant mechanism. These results are consistent with those of Bell et al. (2012) ▶ who reported similar antioxidant effects with the bark extract of *B. orellana* in the rat liver (Bell et al., 2012 ▶). Based on this information, we suggest that this activity may happen through glutathione reductase activation and glutathione peroxidase inhibition, or lipid peroxidation inhibition as demonstrated by decreased MDA concentrations and increased GSH values.

The mechanisms of APAP hepato-toxicity are well described, and the role of inflammation in initiating and propagating liver injury was highlighted by evidence (Sabir and Rocha, 2008 ▶). In fact, Kupffer cells generate pro-inflammatory and chemotactic cytokines, such as TNF-α, leading to the infiltration and activation of neutrophils in damaged liver tissues, and therefore, to aggravation of liver injury (Hinson et al., 2010 ▶). Administration of the barks infusion at a dose of 250 mg/kg significantly reduced liver inflammation as evidenced by decreased leukocytes infiltration and TNF-α levels in the liver tissue of treated mice. The decrease of this cytokine suggests that bark’s infusion would interfere with the signaling pathways involved in the inflammatory process. Previous studies on ethanol-induced hepatotoxicity with this plant, reported such anti-inflammatory activity with leaf extracts (Lopez et al., 2017 ▶). Furthermore, the non-significant decrease observed at the dose of 100 mg/kg, can be explained by the fact that at this dose, the active molecules are not sufficient enough to inhibit the inflammatory process. These results are in line with the findings reported by Lee et al. (2019) ▶ who demonstrated that the protective effects of escin on APAP-induced liver injury are mediated by the inhibition of ERK signalling pathway (Lee et al., 2019 ▶). Therefore, the barks infusion of *B. orellana* has been shown in this study as an extract with anti-inflammatory and antioxidant potential in APAP-induced liver damage.

Extract from bark of *B. orellana* is more active than leaves and roots extracts. Moreover, the bark’s infusion of *B. orellana* was a more potent hepatoprotective extract than macerate and decoction, acting mainly on sub-acute APAP liver injury through anti-inflammatory and antioxidant mechanisms. In addition, the activity was comparable with that of silymarin. This work paved the way through the isolation of active ingredients from bark infusion, which will be advantageous to produce new bioactive compounds with greater activity against liver damage. 
